# The ProteomeXchange consortium in 2020: enabling ‘big data’ approaches in proteomics

**DOI:** 10.1093/nar/gkz984

**Published:** 2019-11-05

**Authors:** Eric W Deutsch, Nuno Bandeira, Vagisha Sharma, Yasset Perez-Riverol, Jeremy J Carver, Deepti J Kundu, David García-Seisdedos, Andrew F Jarnuczak, Suresh Hewapathirana, Benjamin S Pullman, Julie Wertz, Zhi Sun, Shin Kawano, Shujiro Okuda, Yu Watanabe, Henning Hermjakob, Brendan MacLean, Michael J MacCoss, Yunping Zhu, Yasushi Ishihama, Juan A Vizcaíno

**Affiliations:** 1 Institute for Systems Biology, Seattle, WA 98109, USA; 2 Center for Computational Mass Spectrometry, University of California, San Diego (UCSD), La Jolla, CA 92093, USA; 3 Department Computer Science and Engineering, University of California, San Diego (UCSD), La Jolla, CA 92093, USA; 4 Skaggs School of Pharmacy and Pharmaceutical Sciences, University of California, San Diego (UCSD), La Jolla, CA 92093, USA; 5 University of Washington, Seattle, WA 98195, USA; 6 European Molecular Biology Laboratory, European Bioinformatics Institute (EMBL-EBI), Wellcome Trust Genome Campus, Hinxton, Cambridge, CB10 1SD, UK; 7 Faculty of Contemporary Society, Toyama University of International Studies, Toyama 930–1292, Japan; 8 Database Center for Life Science (DBCLS), Joint Support-Center for Data Science Research, Research Organization of Information and Systems, Chiba 277–0871, Japan; 9 Niigata University Graduate School of Medical and Dental Sciences, Niigata 951–8510, Japan; 10 State Key Laboratory of Proteomics, Beijing Proteome Research Center, National Center for Protein Sciences (Beijing), Beijing Institute of Life Omics, Beijing 102206, China; 11 Graduate School of Pharmaceutical Sciences, Kyoto University, Kyoto 606–8501, Japan

## Abstract

The ProteomeXchange (PX) consortium of proteomics resources (http://www.proteomexchange.org) has standardized data submission and dissemination of mass spectrometry proteomics data worldwide since 2012. In this paper, we describe the main developments since the previous update manuscript was published in *Nucleic Acids Research* in 2017. Since then, in addition to the four PX existing members at the time (PRIDE, PeptideAtlas including the PASSEL resource, MassIVE and jPOST), two new resources have joined PX: iProX (China) and Panorama Public (USA). We first describe the updated submission guidelines, now expanded to include six members. Next, with current data submission statistics, we demonstrate that the proteomics field is now actively embracing public open data policies. At the end of June 2019, more than 14 100 datasets had been submitted to PX resources since 2012, and from those, more than 9 500 in just the last three years. In parallel, an unprecedented increase of data re-use activities in the field, including ‘big data’ approaches, is enabling novel research and new data resources. At last, we also outline some of our future plans for the coming years.

## INTRODUCTION

Mass spectrometry (MS)-based proteomics approaches are becoming increasingly prominent in the life sciences. Since its inception, the ProteomeXchange (PX) consortium of proteomics resources (1,2) (http://www.proteomexchange.org) has aimed to standardize data submission and dissemination of public MS proteomics data worldwide. The implementation of the PX consortium formally started in 2012 and since then, it has become the *de facto* standard for sharing MS proteomics datasets in the public domain. Thanks to the perceived reliability of PX resources and in parallel, to the requirements of scientific journals and funding agencies, common practice has changed rapidly in the proteomics field and data sharing has become the norm.

The first stable implementation of the PX data workflow (1) started in 2012 and involved two resources at the time: the PRIDE database (3) (European Bioinformatics Institute, EMBL-EBI, Hinxton, UK) and the PASSEL (4) resource within PeptideAtlas (Institute for Systems Biology, Seattle, WA, USA), a specialized repository for SRM (Selected Reaction Monitoring) experiments. Additionally, PeptideAtlas (5) participated as a resource that re-analyzed public submitted datasets. Two additional resources, MassIVE (University of California San Diego, CA, USA) and jPOST (6) (the jPOST project, Japan) joined in 2014 and 2016, respectively, demonstrating the global reach of PX. A common data access portal called ProteomeCentral was also developed (http://proteomecentral.proteomexchange.org), providing the ability to search for datasets in all participating PX resources at once. The submitted dataset files remain in the receiving resources but are then linked from ProteomeCentral. PX resources support and implement the relevant MS related open data formats of the Proteomics Standards Initiative (PSI) (7,8) and develop and maintain open-source software, including several parser libraries and tools to support these data standards (9,10).

All PX resources share the same dataset identifier space (PXD or RPXD identifiers, see http://www.ebi.ac.uk/miriam/main/collections/MIR:00000513). Additionally, resources may also use their own custom identifiers for non-proteomics MS datasets or datasets that do not fully comply with PX guidelines (e.g. MassIVE also supports metabolomics datasets submitted through GNPS (11)), but these are not tracked and announced via PX.

Here we provide an update of the activities of the PX consortium and its individual resources since the previous update paper was published in *Nucleic Acids Research* (NAR) three years ago (2), including a description of the updated submission guidelines, which now reflect PX’s further expansion to six members. We will also highlight different submission statistics to demonstrate the wide adoption of PX, highlight data re-use activities and discuss future developments.

## EXPANSION OF THE CONSORTIUM AND UPDATED SUBMISSION GUIDELINES

All PX receiving resources store MS proteomics data in a place independent from the control of the original data generators, providing private access for reviewers and journal editors during the manuscript review process. See Table 1 for accessing the basic information about each resource. Table 2 provides a summary of the main functionality offered by the PX members. More detailed information is available in Supplementary File 1. The updated submission guidelines, including now all six members, are available at http://www.proteomexchange.org/docs/guidelines_px.pdf.

**Table 1.** Overview of the main characteristics of the current PX resources

Resource Name	Institution, Country	URL	Function in PX	Contact
PRIDE	European Bioinformatics Institute (EMBL-EBI), Cambridge, UK	http://www.ebi.ac.uk/pride	Archival (Universal)	pride-support@ebi.ac.uk
PeptideAtlas	Institute for Systems Biology, Seattle, WA, USA	http://www.peptideatlas.org/	Re-analysis	http://www.peptideatlas.org/feedback.php
PASSEL	Institute for Systems Biology, Seattle, WA, USA	http://www.peptideatlas.org/passel/	Archival (Focused)	http://www.peptideatlas.org/feedback.php
MassIVE	University of California, San Diego, CA, USA	https://massive.ucsd.edu/	Archival (Universal), Re-analysis	ccms-web@cs.ucsd.edu
jPOST	The jPOST project, Japan	https://jpostdb.org/	Archival (Universal)	https://repository.jpostdb.org/contact
iProX	National Center for Protein Sciences, Beijing, China	https://www.iprox.org/	Archival (Universal)	iprox@iprox.org
Panorama Public	University of Washington, Seattle, WA, USA	https://panoramaweb.org/public.url	Archival (Focused)	panorama@proteinms.net

**Table 2.** Main functionality offered by the PX resources

Data types/ submission types	PRIDE	PASSEL	MassIVE	jPOST	iProX	Panorama Public	Peptide Atlas
**Types of data access**							
Web interface	Yes	Yes	Yes	Yes	Yes	Yes	Yes
Application Programming Interface	Yes (42)	Yes	Yes		Yes	Yes	Yes
Protocol for file transfer (download/ upload)	FTP, Aspera	FTP	FTP	FTP	HTPP, Aspera	WebDAV	FTP
Reviewer private access	File download	File download	File download, web interface	File download	File download, web interface	File download, web interface	N/A
**General functionality/web visualization**							
Dataset centric view	Yes	Yes	Yes	Yes	Yes	Yes	Yes
Protein centric view across resource		Yes	Yes (23)				Yes
Annotated mass spectra	Yes	Yes	Yes	Yes	Yes	Yes	Yes
Chromatograms		Yes				Yes	
Support for RPXD datasets			Yes				Yes
**Standalone tools for re-using datasets**							
Data Visualisation	PRIDE Inspector (10)					Skyline (16)	
Data Analysis	PeptideShaker (43)					Skyline	
Online data analysis pipelines			23 different workflows				

Two additional resources have joined the consortium in the last three years: iProX (12) (National Center for Protein Sciences, Beijing, China) in 2017, and Panorama Public (13) (University of Washington, Seattle, WA, USA) in 2018. iProX is a *universal* data resource (see Section 1 in the submission guidelines for additional details), which can store any type of proteomics datasets, primarily supporting Chinese proteomics researchers. On the other hand, Panorama Public is a *focused* resource, tailored for targeted proteomics approaches.

There are two main data submission workflows, called ‘Complete’ and ‘Partial’. For both submission types, it is mandatory to include a set of common experimental metadata at the level of each dataset (encoded in the shared PX XML format, which is used by ProteomeCentral), raw mass spectra and the submitter's processed results (identification and/or quantification data) are always mandatory in each submitted dataset. In the case of a ‘Complete’ dataset, it is additionally required that the receiving PX resource is able to parse, process and directly connect all individual results with the submitted raw data. This can be achieved if the processed results and the corresponding raw data are available in supported standard data formats like those developed by the PSI (e.g. mzIdentML (14) and mzTab (15)) or in open formats related to Skyline, in the case of Panorama Public.

In contrast, ‘Partial’ datasets contain result files that are not in open standard formats that can be parsed and thus ingested by the receiving repository. The metadata still make the datasets findable in the receiving repository and in ProteomeCentral. In these cases, datasets may then be downloaded and interpreted if suitable software to parse or visualize the files is available. Or the data may be reprocessed using the mandatory raw data as the base. In fact, using the ‘Partial’ dataset submission mechanism, virtually any type of proteomics data workflow is supported.

**Figure 1.**
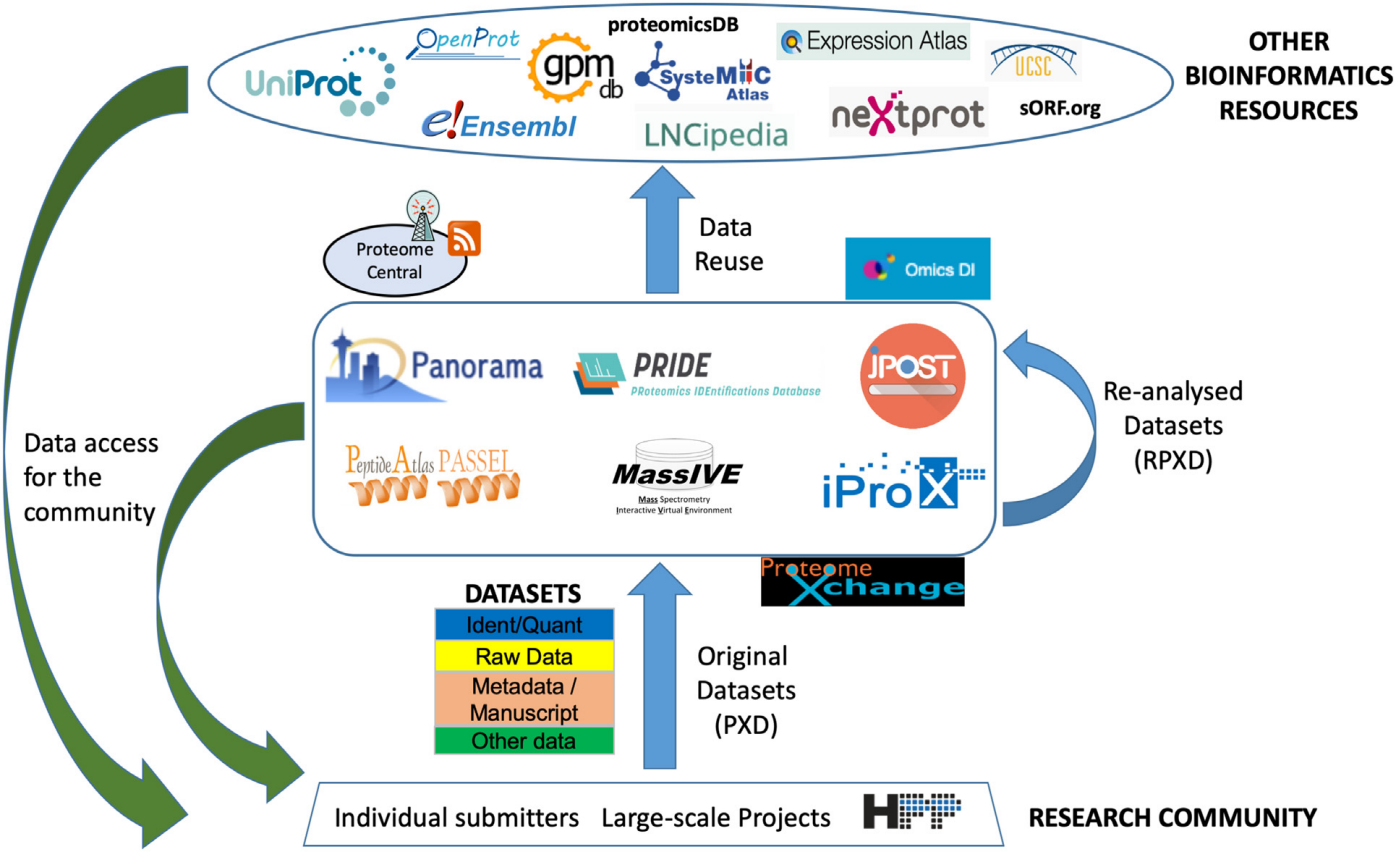
Overview figure including the current ProteomeXchange data workflow and additional bioinformatics resources that are currently re-using proteomics data.

An overview of the current PX resources and data workflows is shown in Figure 1. PRIDE, MassIVE, jPOST and iProX are considered to be *universal* archival resources. At present all of them store datasets mainly coming from DDA (Data Dependent Acquisition) workflows, but they can support other data workflows as well, for instance DIA (Data Independent Acquisition) and top down proteomics, among others, mainly as ‘Partial’ datasets (see submission guidelines for additional details).

PASSEL and Panorama Public, the remaining PX resources, are so-called *focused* resources aimed at targeted proteomics approaches. PASSEL supports any type of SRM data workflows whereas Panorama Public supports results from all quantitative, targeted proteomics workflows enabled by the popular Skyline software ecosystem (16). The other PX members do not actively solicit submission of targeted proteomics data and instead recommend submission to the appropriate focused resources (PASSEL and Panorama Public).

## REPRESENTATION OF REPROCESSED DATASETS AND INTEGRATION WITH OTHER PUBLIC OMICS DATASETS

In addition to original submitted datasets, PX supports the storage and dissemination of re-analyzed versions of datasets that were originally submitted to one of the PX resources. If they are re-analyzed by the PX resources using their own analysis pipelines, these datasets get an identifier of the format RPXD and are also made available through ProteomeCentral, like it happens in the case of PeptideAtlas and MassIVE re-analyzed datasets at present. In all RPXD datasets, the original source of the data is always properly cited and acknowledged with direct links to the original datasets.

As of September 2019, a framework to formalize the representation of different versions of reprocessed datasets by the same PX resource has just been finalized. The corresponding guidelines for reprocessed datasets are available at http://www.proteomexchange.org/docs/reprocessed_guidelines_px.pdf. This functionality is now available via 230 re-analysis *containers* (see e.g. RPXD000669), altogether aggregating 460 re-analyses (e.g. using different algorithms or processing complementary subsets of the data), each of which is associated with an identifier such as RPXD000669.x that combines the RPXD for the container with a number suffix (denoted here as ‘x’) indicating a single re-analysis of the primary data. See all the details in the guidelines for reprocessed datasets.

In our view, appropriate integration of proteomics datasets with other types of public ‘omics datasets is of paramount importance. In this context, datasets in PX resources are included in the OmicsDI (Omics Discovery Index) portal (http://www.omicsdi.org). The objective of OmicsDI is to provide a centralized access point to omics datasets coming from different omics approaches including genomics, transcriptomics, proteomics and metabolomics, linking where possible, multi-omics datasets coming from the same study but available in different resources (17). Recently, OmicsDI has implemented a system to assess the scientific ‘impact’ of omics datasets (18). As a key point, scientists can create their own profiles in OmicsDI, listing their datasets (for instance PX datasets) including several indicators of their scientific impact.

## KEY DEVELOPMENTS IN THE INDIVIDUAL PX RESOURCES

Next, we will highlight some of the recent key developments in the different resources. First of all, PRIDE has been completely redeveloped and a new web and programmatic interfaces are now available. On one hand, this completely new infrastructure supports the increase in the number and volume of submitted datasets and on the other, new features and visualization capabilities. In parallel, PRIDE is increasingly integrating proteomics data in other EMBL-EBI resources (3) such as UniProt (19) (phosphorylation information), Ensembl (20) (proteogenomics) and Expression Atlas (21) (quantitative proteomics experiments).

As explained in Section 3, MassIVE has developed a comprehensive infrastructure for the systematic re-analysis of public datasets, as well as major new developments to aggregate re-analyses into re-usable community knowledge and a new interface for interactive exploration of repository-scale peptide and protein data. First, MassIVE developed new infrastructure for systematic re-analysis of public datasets and applied it to over 30 TBs of human data to derive 364 million new identifications at controlled false discovery rates, all of which are easily accessible at a new simple MassIVE search interface. Second, MassIVE also implemented the concept of publicly accessible re-analyses that are attached to MassIVE datasets (and thus visible whenever the dataset is accessed), as well as the concept of re-analysis *containers* used to group sets of related re-analyses, as mentioned in the previous section. Third, MassIVE capitalized on these large-scale re-analyses to build the largest human spectral library to date (the MassIVE-KB knowledge base (22)), with spectra from over 2 million unique precursor ions covering over 6 million aminoacids from >19 000 canonical human proteins. At last, MassIVE also developed the new Protein Explorer interface (23) to deliver intuitive protein-centric access to hundreds of millions of identifications across datasets, while also cross-referencing MS identifications with functional information from external resources (e.g. UniProt and PhosphoSitePlus).

The jPOST project team has launched a new data journal, *Journal of Proteome Data and Methods* (JPDM, http://www.jhupo.org/jpdm/), to collect precise and detailed metadata. JPDM focuses in both data and metadata and is closely related to jPOST. For example, when users submit their data to the jPOST repository with the minimum required metadata, jPOST provides a template for manuscript submission to JPDM. On one hand, submitters have incentives to improve the FAIRness (Findable, Accessible, Interoperable and Re-useable (24)) of their datasets and publish an additional paper. On the other hand, the repository has the advantage of the additional collected detailed metadata. Once the paper is accepted in JPDM, the metadata will feedback to jPOST. Although this framework was started by the jPOST team, submissions to JPDM are welcome, also where the corresponding datasets are submitted to other PX resources.

## DATA RE-USE ACTIVITIES IN THE FIELD

As a consequence of the unprecedented availability of proteomics data in the public domain, data re-use continues to increase (e.g. (25,26)). Some of the most popular approaches involve the creation of the spectral libraries (22,27), re-analyses activities in the context of the Human Proteome Project ((28), (23) and others), following well-established guidelines (29), and proteogenomics studies, among others. Additionally, it is important to highlight in this context that PX resources are supporting emerging ‘big data’ approaches involving proteomics data, such as machine/deep learning studies. The first studies of this kind are starting to appear in the literature, devoted to e.g. MS/MS spectrum prediction (30–32).

Furthermore, as a key point, external bioinformatics resources to PX are increasingly re-using and integrating proteomics data with other biological data types. Figure 1 highlights some of those resources, including: (i) Protein knowledge bases (UniProt (19) and neXtProt (33)), to support information about each protein's experimental evidence; (ii) Genome browsers (Ensembl (20) and UCSC Genome Browser (34)), where it is possible to integrate proteomics and genomics information using ‘TrackHubs’ (35); (iii) Proteomics resources which re-analyze PX datasets, such as GPMDB (36) and proteomicsDB (37); (iv) EMBL-EBI’s Expression Atlas (21), a resource where protein expression information is increasingly made available together with gene expression data; (v) proteogenomics resources such as LNCipedia (38) and sORF.org (39), where public datasets are routinely reanalyzed to search for evidence of translation for long-non-coding RNAs and short Open Reading Frames, respectively; and (vi) other specialized resources such as SysteMHC Atlas (40) (for immunopeptidomics data) and OpenProt (41). We anticipate that the number of resources re-using public proteomics data will only increase in the near future.

## OVERALL DATA SUBMISSION AND DATA ACCESS STATISTICS

**Figure 2.**
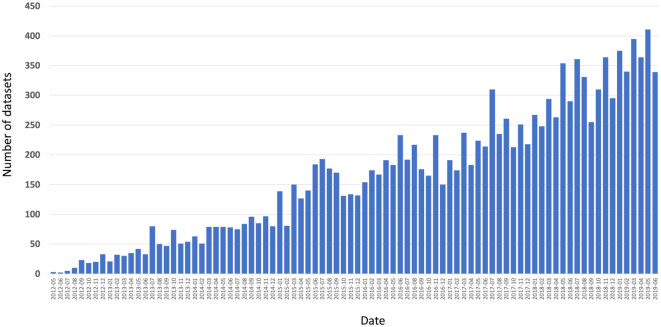
Number of submitted datasets per month to PX resources, ranging from May 2012 to June 2019.

**Figure 3.**
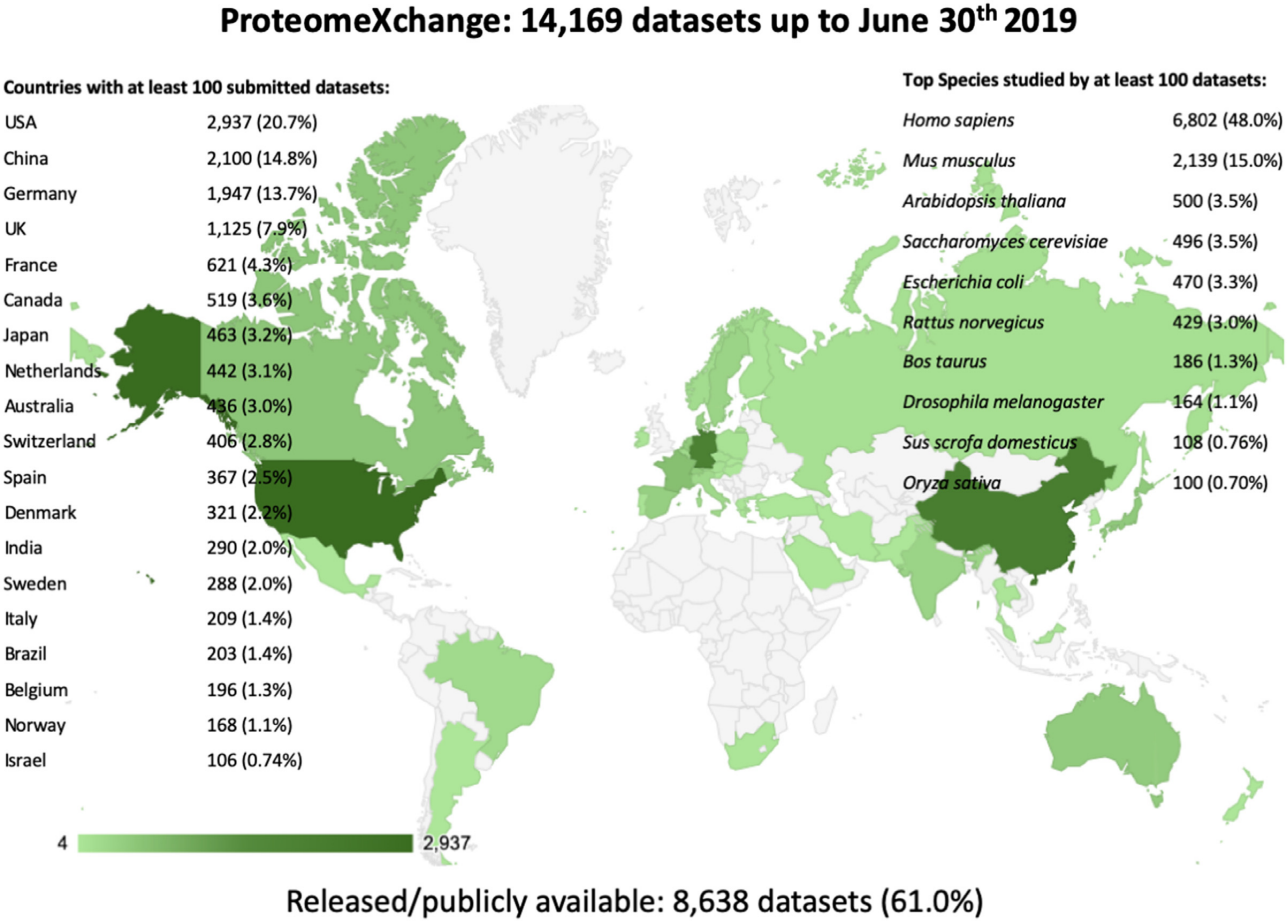
Summary of metrics for datasets available in all ProteomeXchange resources (as of 30 June 2019).

By the end of June 2019, a total of 14 169 datasets had been submitted to PX resources. Of those, 8 638 datasets (61.0%) were already publicly available, whereas the rest were still unreleased (5 531 datasets, 39.0%). Since 2012, the number of submitted datasets has increased substantially every year, a trend that has not stopped yet (Figure 2). If we consider just the last couple of years, during 2017 and 2018, 2 711 and 3 632 datasets were submitted to PX resources, respectively. During the first 6 months of 2019, the number is already 2 224 datasets. In terms of distribution of datasets across individual resources, 12 335 datasets (87.1%), had been submitted to PRIDE, followed by MassIVE (1 126 datasets, 7.9%), jPOST (352 datasets, 2.5%), iProX (174 datasets, 1.2%), PASSEL (139 datasets, 1.0%) and Panorama Public (43 datasets, 0.3%). Datasets come from 50 different countries, demonstrating the global reach of PX. The five most represented countries (Figure 3) are USA (2 937 datasets, 20.7%), China (2 100 datasets, 14.8%), Germany (1 947 datasets, 13.7%), United Kingdom (1 125 datasets, 7.9%) and France (621 datasets, 4.3%). The top five species represented (Figure 3) are: *Homo sapiens* (6 802 datasets, 48.0%), *Mus musculus* (2 139 datasets, 15.0%), *Arabidopsis thaliana* (500 datasets, 3.5%), *Saccharomyces cerevisiae* (496 datasets, 3.5%), and *Escherichia coli* (470 datasets, 3.3%).

PXD accession numbers are now widely used in journal articles to cite datasets. As of August 2019, the number of publications referring to PX datasets in Europe PubMed Central was 5 064, which included the number of occurrences in the abstract for all articles and in the full text for those articles that are open access.

Sustainability of accessible public repositories is a key factor. To ensure long-term data availability, the PX members have committed to importing the data available in any of the other PX resources if the resource has funding problems and must cease its operations. As an additional incentive towards sustainability, data are also routinely replicated across resources to enable systematic re-analyses, as MassIVE currently illustrates with more than 500 datasets already imported from other PX resources (mainly from PRIDE). The combined file size of all PX resources (as of 30 June 2019, including mirrored datasets) was ∼1.3 PBs.

## LICENSING OF DATASETS

So far, licenses for datasets stored in PX resources have not been uniform. However, all PX resources have decided to move towards a default Creative Commons CC0 license as a minimum level in the coming months, making available globally datasets without any restrictions. It is important to highlight that CC0 was already the default existing license for datasets in MassIVE and jPOST. Additionally, PRIDE follows the EMBL-EBI ‘Terms of use’ (https://www.ebi.ac.uk/about/terms-of-use) and iProX has their own data license terms (https://www.iprox.org/page/iproxDataLisence.html). PeptideAtlas/PASSEL and Panorama Public did not have formalized licenses so far.

It should be noted that, once fully formalized, CC0 license can only be ensured for prospective newly submitted datasets, at least for those PX resources that were not enforcing CC0 already. In the near future, all members aim to move to a default CC-BY. In fact, as of September 2019 CC-BY 4.0 is already the default license applied to new datasets submitted to Panorama Public. CC-BY, in addition to the characteristics of the CC0 license, requires attribution to the original data generators/data submitters. The reason for the rest of the partners to not moving directly into CC-BY is that, at present, the legal consequences of using CC-BY for some ‘downstream’ data re-uses are not fully understood. One example that illustrates this is whether a scientist re-using a spectral library produced by one of the PX resources, would be required to give attribution to all original data generators whose datasets where used to build the library.

Independently from this, we want to stress that attribution should always be provided to the original data generators, as it is already happening routinely in the field (i.e. the original publication and/or dataset identifier should be always cited when re-using PX datasets).

## DISCUSSION AND FUTURE PLANS

PX continues to support the open data culture in the field by promoting and enabling the sharing of proteomics data in the public domain. An increasing number of scientific journals mandate submission of the generated datasets accompanying the submitted manuscripts, including the main proteomics journals (*Molecular and Cellular Proteomics*, *Journal of Proteome Research*, *Proteomics*) and journals from the *Nature* and *PLOS* groups, among others. We are happy to work with other journals to support increasingly-strict guidelines for data deposition.

PX resources are committed to comply with the FAIR principles (24) for biological data. We will then follow closely developments in this area, like those led by ELIXIR (https://elixir-europe.org/) in Europe. In this context, a key aspect that we aim to improve in the near future is the annotation of the datasets. The current requirements were set up in 2011 (with minor updates in 2013), reflecting the discussions at the time, involving many key stakeholders in the field. In order to increase re-usability of datasets, we are working towards enabling improvements in metadata annotation by the data submitters, but also *a posteriori* by data curators and other third parties.

We are also currently actively working on the development of a common Application Programming Interface (API) called ProXI to access mass spectra, peptide spectrum matches, peptides, proteins and dataset metadata in a uniform fashion in all PX resources. Additionally, access to mass spectra will be enabled by using the Universal Spectrum Identifier (USI, http://www.psidev.info/usi) system, still under development.

One increasingly relevant topic is the management of clinical, potentially sensitive, proteomics data and whether they should be considered as patient identifiable or not. This topic has recently gained more relevance after the introduction of the GDPR (General Data Protection Regulation) guidelines by the European Union. The proteomics community needs to develop rules and best-practice guidelines for dealing with this type of datasets and, moreover, to evaluate the alignment of these efforts with the genomics and transcriptomics communities. We anticipate that it is likely that some proteomics datasets will need to be controlled-access, so alternative data submission mechanisms will have to be developed for those. At present, authors that have already been advised to follow different data management practices for potentially sensitive proteomics datasets, are advised to contact resources such as EGA (European Genotype-phenome Archive), dbGAP or JGA (Japanese Genotype-phenotype Archive).

It is important to note that the consortium remains open to accept new members. At last, we want to highlight that up to date documentation is linked from the PX website (http://www.proteomexchange.org/). For regular announcements of all the new publicly available datasets, users can follow our Twitter account (@proteomexchange) or subscribe to the following Rich Site Summary (RSS) feed (https://groups.google.com/forum/feed/proteomexchange/msgs/rss_v2_0.xml).

## Supplementary Material

gkz984_Supplemental_File
