# Supplementary material for: The ProteomeXchange consortium in 2020: enabling ‘big data’ approaches in proteomics
Source: Nucleic Acids Res. 2019 Nov 5;48(D1):D1145–52. doi: 10.1093/nar/gkz984 (PMC7145525; doi:10.1093/nar/gkz984)
Supplement: gkz984_Supplemental_File [file gkz984_supplemental_file.docx]

## **Supplementary File 1: Repository resources for data access and reutilization**

## 1. MassIVE, <http://massive.ucsd.edu>

- [Dataset submission](http://proteomics.ucsd.edu/service/massive/documentation/submit-data/)
  - [Main dataset submission page](https://massive.ucsd.edu/ProteoSAFe/index.jsp?params=%7B%22workflow%22:%22MASSIVE-COMPLETE%22%7D) [see [documentation here](http://proteomics.ucsd.edu/service/massive/documentation/submit-data/)]
    - Complete submissions support for
      - Spectrum file formats: mzML (preferred), mzXML, MGF
      - Identification file formats: mzIdentML, mzTab
      - DDA data; example [PXD014834](http://proteomecentral.proteomexchange.org/cgi/GetDataset?ID=PXD014834) / [MSV000079852](https://massive.ucsd.edu/ProteoSAFe/dataset.jsp?task=b218029a9dc9431c87b4104e59d06c4b)
      - DIA data; example [PXD011026](http://proteomecentral.proteomexchange.org/cgi/GetDataset?ID=PXD011026) / [MSV000079200](https://massive.ucsd.edu/ProteoSAFe/dataset.jsp?task=9da2e77c75b243a78620796906e0cfb6)
    - Partial submissions support for all types of untargeted mass spectrometry data
  - Online conversion to standard spectrum formats: [raw and wiff to mzML](https://massive.ucsd.edu/ProteoSAFe/index.jsp?params=%7B%22workflow%22:%22CONVERTRAW%22%7D)
  - Online conversion to standard identification formats: [TSV to mzTab](https://massive.ucsd.edu/ProteoSAFe/index.jsp?params=%7B%22workflow%22:%22CONVERT-TSV%22%7D)
  - Dataset updates:
    - Adding/editing metadata for existing datasets: click “[Add/Update Metadata](http://proteomics.ucsd.edu/service/massive/documentation/add-update-metadata/)” from dataset page
    - Adding citations to datasets: click “[Add Publication](http://proteomics.ucsd.edu/service/massive/documentation/add-publication/)” from dataset page
    - Adding files to existing datasets: click “Add Files” from dataset page
- [Dataset access](http://proteomics.ucsd.edu/service/massive/documentation/access-public-datasets/#MassIVEDatasetBrowsing-Viewing)
  - Data visualization
    - All results files; for example [125 patient samples](https://massive.ucsd.edu/ProteoSAFe/result.jsp?task=120ab12f58594dd29c5a71de529a9686&view=view_result_list)
    - Spectrum identifications; example [38,290 spectra](https://massive.ucsd.edu/ProteoSAFe/result.jsp?task=120ab12f58594dd29c5a71de529a9686&view=group_by_spectrum&file=f.MSV000079852%2Fccms_result%2Fresult%2FColorectalCancer%2FTCGA-AA-3715-01A-22_Proteome_VU_20120821.mzTab) (patient TCGA-AA-3715-01A-22)
    - Peptide identifications; example [13,017 peptides](https://massive.ucsd.edu/ProteoSAFe/result.jsp?task=120ab12f58594dd29c5a71de529a9686&view=group_by_peptide_derived&file=f.MSV000079852%2Fccms_result%2Fresult%2FColorectalCancer%2FTCGA-AA-3715-01A-22_Proteome_VU_20120821.mzTab) (patient TCGA-AA-3715-01A-22)
    - Protein identifications; example [3,422 proteins](https://massive.ucsd.edu/ProteoSAFe/result.jsp?task=364f92fef8e543ba8d1c521a4eb72e7e&view=group_by_protein&file=f.RMSV000000004%2F2018-05-07_ccms_364f92fe%2Fccms_result%2FmzTab%2FCPTAC_Colorectal_Proteomics_-_MSGF__-_TCGA-AA-3715-01A-22_Proteome_VU_20120821_-_50_ppm_precursor_mass_tolerance.mzTab#%7B%22table_sort_history%22%3A%22Variants_dsc%3BPeptides_dsc%22%2C%22accession_input%22%3A%22sp%7C%22%7D) (patient TCGA-AA-3715-01A-22)
    - Requirements for submission to Molecular and Cellular Proteomics
      - Protein one-hit wonders; example [1,467 proteins](https://massive.ucsd.edu/ProteoSAFe/result.jsp?task=364f92fef8e543ba8d1c521a4eb72e7e&view=group_by_protein&file=f.RMSV000000004%2F2018-05-07_ccms_364f92fe%2Fccms_result%2FmzTab%2FCPTAC_Colorectal_Proteomics_-_MSGF__-_TCGA-AA-3715-01A-22_Proteome_VU_20120821_-_50_ppm_precursor_mass_tolerance.mzTab#%7B%22Peptides_lowerinput%22%3A%221%22%2C%22Peptides_upperinput%22%3A%221%22%2C%22accession_input%22%3A%22sp%7C%22%7D) (patient TCGA-AA-3715-01A-22)
      - Post-translational modifications; example [8 modifications](https://massive.ucsd.edu/ProteoSAFe/result.jsp?task=364f92fef8e543ba8d1c521a4eb72e7e&view=group_by_ptm&file=f.RMSV000000004%2F2018-05-07_ccms_364f92fe%2Fccms_result%2FmzTab%2FCPTAC_Colorectal_Proteomics_-_MSGF__-_TCGA-AA-3715-01A-22_Proteome_VU_20120821_-_50_ppm_precursor_mass_tolerance.mzTab#%7B%22table_sort_history%22%3A%22Hits_dsc%22%7D) (patient TCGA-AA-3715-01A-22)
  - Repository-scale queries: [MassIVE search](https://massive.ucsd.edu/ProteoSAFe/massive_search.jsp) (over 450 million PSMs)
  - Protein centric view across resource: [Protein Explorer](https://massive.ucsd.edu/ProteoSAFe/protein_explorer_splash.jsp)
- [Reviewer support](http://proteomics.ucsd.edu/service/massive/documentation/reviewer-access/)
  - Dataset download: FTP
  - Data visualization: same support as for public datasets
  - Data analysis: same as dataset reanalysis (23 different analysis workflows, see “Dataset reanalysis” below)
- [Dataset reanalysis](http://proteomics.ucsd.edu/service/massive/documentation/reanalyze-spectra/)
  - RPXD support:
    - Yes, can [share reanalysis results for any public dataset](http://proteomics.ucsd.edu/service/massive/documentation/share-reanalyses/)
    - For example, see “[Dataset Reanalyses](https://massive.ucsd.edu/ProteoSAFe/dataset.jsp?task=120ab12f58594dd29c5a71de529a9686)” section of dataset page
  - Supported reanalysis formats: mzIdentML, mzTab, TSV (via online conversion to mzTab)
  - Online Data Analysis tools: 23 different analysis workflows at <http://proteomics.ucsd.edu/ProteoSAFe>, including examples below for MassIVE reanalyses of CPTAC patient TCGA-AA-3518-01A-11 (in dataset [PXD014834](https://massive.ucsd.edu/ProteoSAFe/dataset.jsp?task=120ab12f58594dd29c5a71de529a9686))
    - Database Search: [MS-GF+](http://proteomics.ucsd.edu/ProteoSAFe/index.jsp?params=%7B%22workflow%22:%22MSGF_PLUS%22%7D); example [MSGF+ reanalysis](https://proteomics.ucsd.edu/ProteoSAFe/status.jsp?task=cc3bd948861d41f492e95c5bd5b03e3f) (released as [RPXD006619.9](https://massive.ucsd.edu/ProteoSAFe/reanalysis.jsp?task=364f92fef8e543ba8d1c521a4eb72e7e))
    - Spectral Library Search: [MSPLIT](http://proteomics.ucsd.edu/ProteoSAFe/index.jsp?params=%7B%22workflow%22:%22MSPLIT_NEW%22%7D); example [MSPLIT reanalysis](https://proteomics.ucsd.edu/ProteoSAFe/status.jsp?task=335ea50b48494abaaf32c4a8c9d7b753) (released as [RPXD006619.5](https://massive.ucsd.edu/ProteoSAFe/reanalysis.jsp?task=cbf6cb2ef84745aeadd20532089d0adf))
    - Proteogenomics Database Search: [Enosi](http://proteomics.ucsd.edu/ProteoSAFe/index.jsp?params=%7B%22workflow%22:%22ENOSIC2%22%7D); example [Enosi reanalysis](https://proteomics.ucsd.edu/ProteoSAFe/status.jsp?task=de1537c2d0a246c8941ea310b5b5bb35) (released as [RPXD006619.8](https://massive.ucsd.edu/ProteoSAFe/reanalysis.jsp?task=f31a08e233d047e6993080cc23608a6c))
    - Multi-Blind Modification Search: [MODa](http://proteomics.ucsd.edu/ProteoSAFe/index.jsp?params=%7B%22workflow%22:%22MODA%22%7D); example [MODa reanalysis](https://proteomics.ucsd.edu/ProteoSAFe/status.jsp?task=e8ba1b9e30a3466992d57d172399fcd5) (released as [RPXD006619.4](https://massive.ucsd.edu/ProteoSAFe/reanalysis.jsp?task=3dd00bc669ec437c9e9cd9e552dfc232))
    - Multi-Pass Modification Search: [Maestro](http://proteomics.ucsd.edu/ProteoSAFe/index.jsp?params=%7B%22workflow%22:%22MAESTRO%22%7D); example [Maestro reanalysis](https://proteomics.ucsd.edu/ProteoSAFe/status.jsp?task=8ec2ee83bd9746d4975ff839e01c570d) (released as [RMSV000000232.6](https://massive.ucsd.edu/ProteoSAFe/reanalysis.jsp?task=c53354d5e15740919af3d7cf233d9152))

## 2. PRIDE, <https://www.ebi.ac.uk/pride/>

- Dataset submission: PX submission tool (Java standalone tool). (<https://www.ebi.ac.uk/pride/help/archive/submission>) (1,2).
  - Support for Complete and Partial submissions (all details here (3,4)).
  - Protocol and post-submission steps (1).
- Dataset access:
  - Web interface (3)
  - Programmatic interface REST (5).
  - File repository: access FTP and Aspera (faster file transfer protocol) (3,4).
  - PRIDE Inspector (6,7).
  - Private access to reviewers via FTP and Aspera (via PRIDE Inspector).
- Support for PSI data standards:
  - Parser libraries/writers:
    - jmzML library (8) (<https://github.com/PRIDE-Utilities/jmzml>).
    - jmzIdentML library (9) (<https://github.com/PRIDE-Utilities/jmzIdentML>).
    - jmzTab (10) (<https://github.com/PRIDE-Utilities/jmzTab>).
    - jmzReader (11) (<https://github.com/PRIDE-Utilities/jmzReader>).
    - ms-data-core-api (12) (<https://github.com/PRIDE-Utilities/ms-data-core-api>).
  - Visualization tool:
    - PRIDE Inspector (6,7).
- Other libraries supporting data submission/access:
  - OLS client and OLS Dialog (13).
- Sister resource for spectral clustering results across PRIDE:
  - PRIDE Peptidome (formally known as PRIDE Cluster) (14,15).
- Data re-analysis/dissemination:
  - Analysis tool connected to PRIDE (Compomics team) -> PeptideShaker (16).
  - Data dissemination to UniProt (3) (pre-print: <https://doi.org/10.1101/541656>).
  - Data dissemination to Ensembl (proteogenomics data) (3).
  - Data dissemination to EBI’s Expression Atlas (quantitative proteomics) (3,17) (pre-print: <https://doi.org/10.1101/665968>).

## 3. Panorama Public,<https://panoramaweb.org/public.url>

- Dataset Submission
  - Panorama Public has support for “complete” submissions of targeted mass spectrometry results processed and analyzed with Skyline.
  - Submitting data to Panorama Public is documented here: [Submit Data to Panorama Public](https://panoramaweb.org/doc_panorama_public.url)
  - In addition to uploading Skyline documents and raw data, submitters can also include other supplementary information such as tables and figures with their submission. This is documented in a tutorial: [Submitting Supplementary Data to Panorama Public](https://panoramaweb.org/tutorial_panorama_public.url)
  - Submitters have read-only access to their data in Panorama Public. Any changes required, to address reviewer feedback for example, can be made in the submitters’ space on [PanoramaWeb](https://panoramaweb.org/wiki/home/page.view?name=publish_to_panorama_public) and re-submitted to Panorama Public by clicking the “[Resubmit](https://panoramaweb.org/wiki/home/page.view?name=publish_to_panorama_public#resubmit)” button.
- Dataset Access
  - Data visualization
    - Browse the list of analytes targeted in Skyline documents. Filter and export results in several formats
    - View extracted chromatograms with annotated peak boundaries (requirement for [submission to Molecular and Cellular Proteomics](http://www.mcponline.org/site/misc/MCP_Targeted_Mass_Spec_Guidelines_1.27.17.pdf)).
      - Example: [PXD014474](http://proteomecentral.proteomexchange.org/cgi/GetDataset?ID=PXD014474) /<https://panoramaweb.org/ovarian_cancer_biomarker.url>
        - Peptide [DIENFDSTQK](https://panoramaweb.org/targetedms/Panorama%20Public/2019/UCSF%20Krogan%20Lab%20-%20Ovarian%20cancer%20biomarker/showPeptide.view?id=7725672)
    - Protein sequence coverage by peptide targets measured in a Skyline document.
    - Annotated MS/MS spectra from spectrum libraries associated with Skyline documents.
    - Summary charts for comparing peak areas and retention times across replicates in a Skyline document.
    - View additional supplementary information provided by submitters in the form of figures or tables.
      - Example: [PXD011297](http://proteomecentral.proteomexchange.org/cgi/GetDataset?ID=PXD011297) /<https://panoramaweb.org/singlepointcal.url>
  - A search interface is available to query the repository for datasets containing specific proteins and peptides, as well as by instrument and organism.
  - Skyline documents available on Panorama Public can be downloaded for further exploration with the Skyline software for targeted proteomics. Researchers can use these documents as a guide to facilitate their proteomics investigations with Skyline or to reproduce published experimental results. They can also be used as functioning template documents with a set of targets that may be useful for future research.
  - In addition to accessing and downloading data in a web browser, Panorama Public supports data downloads via the [WebDAV](https://en.wikipedia.org/wiki/WebDAV) protocol. This can be done by using a WebDAV client such as [CyberDuck](https://cyberduck.io/), [WinSCP](https://winscp.net/eng/docs/introduction), or by using “Map network drive..”' in the Windows File Explorer.
- Reviewer Support
  - Same support as for public datasets for data visualization and download.

## 4. jPOST, <https://jpostdb.org/>

Detailed information can be found in the following publications (18,19), and in the jPOST documentation pages (<https://repository.jpostdb.org/help>).

## 5. iProX, [https://www.iprox.org/](http://www.iprox.org/)

Detailed information can be found in the following publication (20), and in the iProX documentation pages (<https://www.iprox.org/page/helpEn.html>).

6. PASSEL, <http://www.peptideatlas.org/passel/>

Detailed information can be found in the following publication (21,22), and in the PASSEL documentation pages (<http://www.peptideatlas.org/passel/>).

7. Peptide Atlas, <http://www.peptideatlas.org/>

Detailed information can be found in the following publication (22), in the different publications including the Peptide Atlas builds coming from different species (<http://www.peptideatlas.org/builds/>) and in the Peptide Atlas documentation pages (<http://www.peptideatlas.org/overview.php>).

**References**

- - 1. Ternent, T., Csordas, A., Qi, D., Gomez-Baena, G., Beynon, R.J., Jones, A.R., Hermjakob, H. and Vizcaino, J.A. (2014) How to submit MS proteomics data to ProteomeXchange via the PRIDE database. *Proteomics*, **14**, 2233-2241.
  - 2. Jarnuczak, A.F. and Vizcaino, J.A. (2017) Using the PRIDE Database and ProteomeXchange for Submitting and Accessing Public Proteomics Datasets. *Curr Protoc Bioinformatics*, **59**, 13 31 11-13 31 12.
  - 3. Perez-Riverol, Y., Csordas, A., Bai, J., Bernal-Llinares, M., Hewapathirana, S., Kundu, D.J., Inuganti, A., Griss, J., Mayer, G., Eisenacher, M. *et al.* (2019) The PRIDE database and related tools and resources in 2019: improving support for quantification data. *Nucleic acids research*, **47**, D442-D450.
  - 4. Vizcaino, J.A., Csordas, A., del-Toro, N., Dianes, J.A., Griss, J., Lavidas, I., Mayer, G., Perez-Riverol, Y., Reisinger, F., Ternent, T. *et al.* (2016) 2016 update of the PRIDE database and its related tools. *Nucleic acids research*, **44**, D447-456.
  - 5. Reisinger, F., Del-Toro, N., Ternent, T., Hermjakob, H. and Vizcaino, J.A. (2015) Introducing the PRIDE Archive RESTful web services. *Nucleic acids research*.
  - 6. Wang, R., Fabregat, A., Rios, D., Ovelleiro, D., Foster, J.M., Cote, R.G., Griss, J., Csordas, A., Perez-Riverol, Y., Reisinger, F. *et al.* (2012) PRIDE Inspector: a tool to visualize and validate MS proteomics data. *Nat Biotechnol*, **30**, 135-137.
  - 7. Perez-Riverol, Y., Xu, Q.W., Wang, R., Uszkoreit, J., Griss, J., Sanchez, A., Reisinger, F., Csordas, A., Ternent, T., Del-Toro, N. *et al.* (2016) PRIDE Inspector Toolsuite: Moving Toward a Universal Visualization Tool for Proteomics Data Standard Formats and Quality Assessment of ProteomeXchange Datasets. *Mol Cell Proteomics*, **15**, 305-317.
  - 8. Cote, R.G., Reisinger, F. and Martens, L. (2010) jmzML, an open-source Java API for mzML, the PSI standard for MS data. *Proteomics*, **10**, 1332-1335.
  - 9. Reisinger, F., Krishna, R., Ghali, F., Rios, D., Hermjakob, H., Vizcaino, J.A. and Jones, A.R. (2012) jmzIdentML API: A Java interface to the mzIdentML standard for peptide and protein identification data. *Proteomics*, **12**, 790-794.
  - 10. Xu, Q.W., Griss, J., Wang, R., Jones, A.R., Hermjakob, H. and Vizcaino, J.A. (2014) jmzTab: a java interface to the mzTab data standard. *Proteomics*, **14**, 1328-1332.
  - 11. Griss, J., Reisinger, F., Hermjakob, H. and Vizcaino, J.A. (2012) jmzReader: A Java parser library to process and visualize multiple text and XML-based mass spectrometry data formats. *Proteomics*, **12**, 795-798.
  - 12. Perez-Riverol, Y., Uszkoreit, J., Sanchez, A., Ternent, T., Del Toro, N., Hermjakob, H., Vizcaino, J.A. and Wang, R. (2015) ms-data-core-api: an open-source, metadata-oriented library for computational proteomics. *Bioinformatics*, **31**, 2903-2905.
  - 13. Perez-Riverol, Y., Ternent, T., Koch, M., Barsnes, H., Vrousgou, O., Jupp, S. and Vizcaino, J.A. (2017) OLS Client and OLS Dialog: Open Source Tools to Annotate Public Omics Datasets. *Proteomics*, **17**.
  - 14. Griss, J., Perez-Riverol, Y., Lewis, S., Tabb, D.L., Dianes, J.A., Del-Toro, N., Rurik, M., Walzer, M.W., Kohlbacher, O., Hermjakob, H. *et al.* (2016) Recognizing millions of consistently unidentified spectra across hundreds of shotgun proteomics datasets. *Nat Methods*, **13**, 651-656.
  - 15. Griss, J., Foster, J.M., Hermjakob, H. and Vizcaino, J.A. (2013) PRIDE Cluster: building a consensus of proteomics data. *Nat Methods*, **10**, 95-96.
  - 16. Vaudel, M., Burkhart, J.M., Zahedi, R.P., Oveland, E., Berven, F.S., Sickmann, A., Martens, L. and Barsnes, H. (2015) PeptideShaker enables reanalysis of MS-derived proteomics data sets. *Nat Biotechnol*, **33**, 22-24.
  - 17. Papatheodorou, I., Fonseca, N.A., Keays, M., Tang, Y.A., Barrera, E., Bazant, W., Burke, M., Fullgrabe, A., Fuentes, A.M., George, N. *et al.* (2018) Expression Atlas: gene and protein expression across multiple studies and organisms. *Nucleic acids research*, **46**, D246-D251.
  - 18. Okuda, S., Watanabe, Y., Moriya, Y., Kawano, S., Yamamoto, T., Matsumoto, M., Takami, T., Kobayashi, D., Araki, N., Yoshizawa, A.C. *et al.* (2017) jPOSTrepo: an international standard data repository for proteomes. *Nucleic acids research*, **45**, D1107-D1111.
  - 19. Moriya, Y., Kawano, S., Okuda, S., Watanabe, Y., Matsumoto, M., Takami, T., Kobayashi, D., Yamanouchi, Y., Araki, N., Yoshizawa, A.C. *et al.* (2019) The jPOST environment: an integrated proteomics data repository and database. *Nucleic acids research*, **47**, D1218-D1224.
  - 20. Ma, J., Chen, T., Wu, S., Yang, C., Bai, M., Shu, K., Li, K., Zhang, G., Jin, Z., He, F. *et al.* (2019) iProX: an integrated proteome resource. *Nucleic acids research*, **47**, D1211-D1217.
  - 21. Farrah, T., Deutsch, E.W., Kreisberg, R., Sun, Z., Campbell, D.S., Mendoza, L., Kusebauch, U., Brusniak, M.Y., Huttenhain, R., Schiess, R. *et al.* (2012) PASSEL: the PeptideAtlas SRMexperiment library. *Proteomics*, **12**, 1170-1175.
  - 22. Kusebauch, U., Deutsch, E.W., Campbell, D.S., Sun, Z., Farrah, T. and Moritz, R.L. (2014) Using PeptideAtlas, SRMAtlas, and PASSEL: Comprehensive Resources for Discovery and Targeted Proteomics. *Curr Protoc Bioinformatics*, **46**, 13 25 11-28.
